# Global genetic carrier testing: a vision for the future

**DOI:** 10.1186/s13073-015-0204-9

**Published:** 2015-07-29

**Authors:** Arthur L. Beaudet

**Affiliations:** Department of Molecular and Human Genetics, Baylor College of Medicine, Houston, TX 77030 USA

## Abstract

Expanded genetic carrier testing is changing clinical practice. Current experience highlights the need for rigorous curation of tens of thousands of variants as to their pathogenicity and phenotypic effects. There is an urgent need for strategies to present a range of options to families to enable them to make informed decisions. The potential exists to avoid the great majority of serious inherited, but not de novo, single-gene disabilities.

## Rapid proliferation

For decades, genetic carrier testing has referred primarily to testing with the aim of identifying heterozygote carriers of mutations that put a mating of two carriers at a one in four risk of having a child with a disorder causing serious disability or early death. This type of testing began in the early 1970s with Tay-Sachs disease in the Ashkenazi Jewish population [1]. Screening programs focused on β-thalassemia were implemented in the Mediterranean region around 1980, as well as some programs for sickle cell anemia. The identification of the cystic fibrosis gene in 1989 [2] led to immediate implementation of carrier testing for this disease.

By 1993 [3], the term 'universal carrier testing' was used in the context of cystic fibrosis to imply testing the entire population regardless of ethnicity or family history. In 2010, the biotechnology company Counsyl, Inc. proposed 'universal carrier testing' to mean testing more than 100 genes and talked of “the long tail of Mendelian disease”, implying the possibility of testing for all Mendelian diseases even if relatively rare [4]. Although Counsyl used genotyping technology, Bell et al. in 2011 [5] were the first group to use next-generation sequencing (NGS) to analyze 437 target genes. Since then, there has been a rapid proliferation of expanded carrier testing programs, and it would be only a small step further to use whole-exome or whole-genome sequencing for carrier detection.

Providers are switching to full sequencing of all coding exons and associated regions known to harbor pathogenic mutations. This increased activity forces us to consider how to analyze the data, and we propose the creation of a triple-global database: global first to include all human populations, global second to include the entire genome, and global third to include the most complete understanding possible of variant-to-phenotype relationships. The Human Variome Project [6] and the ClinVar database [7] are efforts in this direction. The phenotypic variation associated with each variant must be determined as distinct from pathogenicity. For example, there might be equally high confidence of pathogenicity for two variants in *HEXA*, but one may cause infantile Tay-Sachs while another causes an adult chronic phenotype. The phenotypic effect will often be the result of two variants in a compound heterozygous state. One source for this global database could be to determine the disease-causing mutations in the majority of individuals across the globe with severe autosomal recessive or X-linked disorders.

## Current practice

Although the term 'carrier' is most often used to designate individuals with heterozygous recessive mutations in autosomal genes, the term can also be used to describe carriers of mutations in X-linked genes, carriers of copy-number variants (CNVs), carriers of dominant mutations such as those in *BRCA1* and *BRCA2* (which are associated with the risks of breast and ovarian cancer), carriers of common single nucleotide polymorphisms (SNPs), and carriers of balanced chromosomal translocations.

At present, carrier testing most commonly involves a gene panel with dozens to hundreds of genes. The disorders most widely screened for are cystic fibrosis, diseases associated with Ashkenazi ethnicity, hemoglobinopathies, spinal muscular atrophy, and fragile X syndrome. In addition to NGS, various forms of copy number analysis are used to detect large deletions and duplications, such as those relevant for Duchenne muscular dystrophy and *NPHP1-*caused nephronophthesis, as well as *MECP2* and *SMN1* duplications and other CNVs.

A recent statement on expanded carrier screening from multiple professional organizations provides an overview of current practices [8]. This statement covers many important topics, which cannot be covered in the space available here, including residual risk, relationship with newborn screening, the difficulties presented by pseudogenes, screening gamete donors, the importance of knowing paternity, and the detection of milder or incompletely penetrant variants and disorders.

Many genes currently being tested relate to severe phenotypes for which therapy is inadequate. Diseases such as Tay-Sachs, Krabbe, and fragile X syndrome are examples for which strong justification exists. Cystic fibrosis and congenital deafness are also often considered in this severe disease group, although they do not involve intellectual disability and some good treatments are available. Screening for these disorders is usually predicated on the expectation that at least some families will want to avoid the birth of affected children, either through termination of affected pregnancies or perhaps through the use of preimplantation genetic diagnosis (PGD) or donor gametes if risk is identified prior to conception. However, many other disorders for which carrier testing seems less compelling but perhaps not harmful are being tested, including phenylketonuria and medium-chain acyl-CoA dehydrogenase (MCAD) deficiency; these are disorders for which newborn screening and excellent treatments are available. Other disorders, such as hereditary fructose intolerance, Wilson disease, and glucose-galactose malabsorption, are usually not detected by newborn screening, and early and accurate diagnosis can be extremely beneficial and even life-saving.

## One vision for the future

The trend towards expanded screening is likely to grow, with a longer and longer list of genes being analyzed in an attempt to address the “long tail of Mendelian disease”. The need for correct interpretation of the data provided by these sequencing approaches emphasizes the usefulness of a triple-global database of human gene variation, as noted above. The development and refinement of this database will be a mammoth task, but it need only be done once per human civilization, and the effort required for its maintenance and updating will be trivial by comparison with that required for its initial construction.

If we were to use whole-genome or whole-exome sequencing as a carrier test, the burden of severe Mendelian disabilities in the population could be dramatically reduced. The incidence of individual recessive disorders varies widely across the globe, presumably largely related to founder effects and some selection for heterozygote advantage. Apart from the data on globin disorders collected by the World Health Organization [9], the combined incidence of all recessive disorders and the incidence of individual disorders are generally unknown for most of the very large country-level populations, such as those in China, India, and Indonesia. The incidence of recessive disorders is higher in populations in which consanguinity is common. If health care systems across the globe supported carrier detection for recessive and dominant variants and funded the use of PGD to avoid serious disabilities, the tools that are already available could dramatically reduce the frequency of inherited, but not de novo*,* cases of severe Mendelian disabilities without a single abortion. One criticism will be that this is prohibitively expensive, but the burden of severe Mendelian disease is also very expensive for society. For dominant disorders that occur mostly through inherited rather than de novo mutations (e.g., *BRCA1*/*2* and *HNPCC* mutations that lead to hereditary nonpolyposis colon cancer), such an approach would dramatically reduce the frequency of these disorders in a single generation and for all generations to come except for new mutations.

For defining pathogenicity of each variant, one option might be as follows: score all inactivating (loss-of-function) variants as 'carrier (pathogenic)' if specific exclusions are made for exceptions (e.g., stop codons in last exons or alternatively spliced exons); score missense variants with appropriate frequency and no contrary evidence as 'carrier (pathogenic)' if the variant was found *in trans* with pathogenic variants (ITWPV) in at least five affected families; score missense variants as 'carrier, moderate evidence (likely pathogenic)' if the variant was detected ITWPV in three or four affected families and as 'carrier, weak evidence' (equivalent to 'variant of unknown significance-favor pathogenic' [VUS-FP]; a term suggested by Heidi Rehm, personal communication) if the variant was detected ITWPV in one or two affected families; score missense variants that have never been reported to cause disease as VUS and do not report these. This strategy ignores computational assessments of pathogenicity, although this situation could change if these tools became more predictive. Many variants reported to be pathogenic in one or two families and described as 'likely pathogenic' in various databases are almost certainly benign. A major effort is needed to reclassify such variants properly. Each variant should also be given a phenotypic score, which in most cases will be 'classic or most severe' associated with homozygous or hemizygous loss-of-function variants or with well-characterized missense variants (e.g., sickle cell), but many variants are associated with milder phenotypes which must be explained when known.

Individuals with a variant identified as 'carrier, weak evidence' or 'carrier, moderate evidence' present a dilemma. If these variants are reported, a couple might be falsely identified as being at risk of having affected offspring, whereas if the variants are not reported there is risk of identifying a couple as not at risk and then having the birth of an unanticipated affected child. The second harm seems greater than the first. In most cases, the partner of the possible carrier will be a noncarrier and the concern can be dropped. Rarely, the partner will be a carrier and special efforts such as functional studies could be undertaken. If the data are worrisome enough, a couple would always have the option to avoid the ambiguous genotype via PGD or termination of pregnancy.

There is a desire on the part of some clinicians and patients to have an option to exclude disorders that are unlikely to alter reproductive plans and to exclude variants with very mild phenotypes and/or only weak evidence of pathogenicity, versus a second option to test and report variants as comprehensively as possible. An option restricted to variants associated with more severe phenotypes can be offered. Some variants that are common in various subpopulations across the globe especially raise questions about whether they should be reported or not. These include milder variants in *G6PD*; homozygous or heterozygous deletion of one of the α-globin genes but not of the *cis* copy; the S and Z variants for α-1-antytrypsin; variants in *CFTR* causing congenital bilateral absence of the vas deferens; and the mildly deleterious 5T allele of the poly-T tract variation in intron 8 of *CFTR*. Reporting these variants or not dramatically affects the fractions of individuals who will have a positive test report.

## Conclusion

With increasing breadth of testing, concurrent testing of both partners is far superior to sequential testing, as the reproductive risks are determined by the combined data. Also, expansion of the gene list means that an increasing proportion of tested individuals will be a carrier for one or other disorder leading to sequential testing of the partner. Undue anxiety and inefficiency can be avoided by concurrent testing. Preconception testing is far superior to testing during pregnancy, as options such as PGD are available. Testing for X-linked disorders and for dominant mutations should be given increasing attention going forward. Carrier testing for pathogenic CNVs of varying severity and penetrance should also be an option (e.g., deletions and duplications of the DiGeorge and Williams syndrome regions). Cascade testing, defined as prioritized testing of individuals on the basis of degree of relatedness to a known carrier [10], is highly desirable and is made more effective by testing both partners. Cascade testing is particularly valuable for dominant mutations such as those in *BRCA1*/*2* or in *HNPCC* genes. One option might be to include testing for so-called actionable genes [11], such as the gene encoding LDL receptor (LRLR). If one envisions exome sequencing or genome sequencing of couples prior to reproduction, one could also include more general forms of testing such as a pharmacogenetic panel.

In summary, expanded carrier testing is medically justifiable, but involves challenges in genetic counseling and great complexity in the interpretation of the pathogenicity and phenoytypic effect of each variant. Testing can be restricted to reproductive decisions or expanded to include actionable gene analysis. A massive and publicly accessible database will be needed to maximize benefit and minimize harms of expanded carrier testing. Expanded carrier testing makes it potentially feasible to avoid the great majority of serious inherited, but not de novo, single-gene disabilities.
